# Evaluating the Impact of Heat Stress on Placental Function: A Systematic Review

**DOI:** 10.3390/ijerph21081111

**Published:** 2024-08-22

**Authors:** Jazmin D. Ramirez, Isabel Maldonado, Katharine J. Mach, Jonell Potter, Raymond R. Balise, Hudson Santos

**Affiliations:** 1 School of Nursing and Health Studies, University of Miami, Coral Gables, FL 33146, USA; icm16@miami.edu (I.M.); hsantos@miami.edu (H.S.); 2 Department of Environmental Science and Policy, Rosenstiel School of Marine, Atmospheric, and Earth Science, University of Miami, Miami, FL 33149, USA; kmach@miami.edu; 3 Leonard and Jayne Abess Center for Ecosystem Science and Policy, University of Miami, Coral Gables, FL 33146, USA; 4 Department of Obstetrics, Gynecology and Reproductive Sciences, University of Miami Leonard M. Miller School of Medicine, Miami, FL 33136, USA; jpotter2@med.miami.edu; 5 Department of Public Health Sciences, University of Miami Leonard M. Miller School of Medicine, Miami, FL 33136, USA; balise@miami.edu

**Keywords:** climate, placenta, childbirth, pregnancy, methods

## Abstract

Ambient heat stress poses a significant threat to public health, with rising temperatures exacerbating the risks associated with pregnancy. This systematic review examined the associations between heat stress exposure and placental function, synthesizing methodologies from the existing literature to inform future research approaches. Analyzing 24 articles, it explores various study designs, temperature exposure parameters, pregnancy windows, and placental outcome variables. Findings across human and animal studies reveal diverse effects on placental weight, efficiency, blood flow, anatomy, gene expression, and steroid levels under heat stress conditions. While animal studies primarily utilize randomized controlled trials, human research relies on observational methodologies due to ethical constraints. Both demonstrate alterations in placental morphology and function, underscoring the importance of understanding these changes for maternal and fetal health. The review underscores the urgent need for further research, particularly in human populations, to elucidate mechanisms and develop interventions mitigating heat stress’s adverse effects on placental health. Ultimately, this synthesis contributes to understanding the complex interplay between environmental factors and pregnancy outcomes, informing strategies for maternal and fetal well-being amidst climate change challenges.

## 1. Introduction

Ambient heat stress is a significant and increasing threat to the health and well-being of societies. Exposure to heat is a major public health concern that is becoming more dire [1]. According to the National Centers for Environmental Information’s annual global report, Earth’s temperatures are increasing every year with the rate of warming doubling each decade since 1981 [2]. Climate change has raised average temperatures as well as the frequency, intensity, and duration of heat events [3]. Urban heat island effects, a phenomenon attributed to urban construction, lack of vegetation coverage, and population density, can compound the consequences of global warming and some regions, particularly those already grappling with persistent extreme temperatures, are likely to exceed their adaptability thresholds [4,5,6,7]. Hundreds of millions of people across multiple climate zones are likely to face heat exposure levels beyond physiological limits [6]. Temperatures exceeding 35 °C (95 °F) in humid surroundings pose a risk to maintaining physiological equilibrium, affecting both core body temperature and hydration levels [8]. Further, rapid and persistent increases in external ambient heat can compromise internal heat regulation and can increase the risk of heat cramps, dehydration, heat exhaustion, and hyperthermia [9]. Heat stress also impacts the body through multiple mechanisms, such as exacerbating cardiovascular disease, respiratory diseases, and mental illness [10,11].

Several populations are known to be disproportionately harmed by heat-related health impacts, including the elderly, those living in poverty, young children, minorities, outdoor workers, individuals with chronic conditions (e.g., cardiovascular and respiratory disorders), socially isolated individuals, those living in already chronically hot locations, and pregnant women [12,13,14]. Pregnant individuals are a particularly vulnerable population to the effect of heat stress on the body and its effects on birth and pregnancy outcomes and continue to be understudied in heat-health research. Heat stress affects maternal health through several mechanisms. During pregnancy, physiological and anatomical changes impact a woman’s ability to thermoregulate [15]. Lower ratios of body surface area to body mass due to weight gain make it harder to dissipate heat and an increase in metabolic demands of the growing fetus increases core body temperatures and susceptibility to dehydration [16]. As established in previous reviews of epidemiological studies, there are associations between ambient heat exposure and pregnancy outcomes, such as preterm birth, low birth weight, and stillbirth, but little is known about how these biological mechanisms are impacted by heat [12,13,14,17].

One mechanism by which heat stress may affect pregnancy and birth outcomes is through the dysregulation of placental function. The placenta’s function is to supply nutrients and blood to the fetus, remove wastes, and produce hormones and it acts as an active and passive barrier to maternal fetal immunological activity [18]. This unique organ has two independent vascular systems where the oxygen content is balanced between fetal and maternal circulations. Variations in one or both vascular systems can disrupt the balance of placental oxygen supply and demand, leading to a decrease in uteroplacental blood flow and hypoxia [19]. Normothermia is maintained during heat exposure by sweating and redirecting blood flow from the visceral organs to the skin, resulting in competition for available cardiac output (the volume of blood the heart pumps per minute, a critical parameter for cardiovascular physiology) [13]. The placenta is heavily reliant on cardiac output for adequate perfusion of oxygen and glucose and adequate substrate concentrations to the fetus [20,21]. During heat stress, placental perfusion may become compromised [22]. Chronic reduction in uteroplacental blood flow can negatively impact fetal health and development, as exhibited by fetal growth restriction and low birth weight [23]. In addition to poor physical outcomes from heat exposure, preliminary findings suggest that intrauterine heat stress has negative effects on the placenta at the molecular level [8]. In a narrative review by Cowell et al., heat stress was linked to fetal and placental growth outcomes in humans, ruminants, and murine species, with a focus on biological pathways that affect the placenta [24]. However, it remains unclear how heat stress affects human placental function.

The methods of this review included a two-part process guided by the aims. First, we searched PubMed to identify studies on heat stress and human placentas. This search yielded several studies with animal models and only one with human subjects, which were imported into Covidence for comparison of methodologies (Aim 1). The second portion of this review addresses the current methodologies of human studies. Epidemiological studies have been extensively reviewed for associations with temperature and birth outcomes and are more recently starting to compare methodologies and study characteristics [14,17,24,25,26,27]. We searched the existing reviews that provide relevant information to describe the methodologies for measuring heat stress during human pregnancy and cross-referenced their citations to conduct a gray literature search of any experimental or observational studies that the reviews may have excluded (Aim 2). We summarized the findings related to methodologies of human study designs, temperature measurement methods, and the pregnancy window of exposure using a narrative review approach. This process identified six scoping or systematic reviews related to heat stress and human pregnancy (Table 1 below).

Of the scoping and systematic reviews, Syed et al. and Bekkar et al. discussed the methodologies in the greatest detail and reviewed a total of *n* = 83 and *n* = 68 studies, respectively, for their methodologies [14,26]. The most common study design was cohort studies (*n* = 31) while the least common was case series (*n* = 2). The reported sample size for the cohort studies ranged from 138 to 56 million subjects and the authors obtained data from birth certificates, death certificates, population registries, and electronic medical records (EMRs). For most of these large cohort studies, the duration of data collection was 5–9 years (28 studies); the second most common duration was less than 5 years (22 studies). The maximum duration of data collection was 25+ years for seven studies [14]. The cohort studies involve temperature data collection at several time points throughout pregnancy, including hourly, daily, monthly, seasonally, based on climate, and based on heat waves. The pregnancy exposure periods included the first trimester, second and third trimesters, all/any part of pregnancy, and the delivery date. Of the cohort studies among all the reviews, preterm birth was the most evaluated pregnancy outcome, followed by birthweight, congenital anomalies, and stillbirths [14].

**Human Observational Studies**: We conducted a literature review of the observational studies cited in these scoping and systematic reviews to conduct a forward search from the reference list. The purpose of searching the references was to cross-reference the citations with the ones yielded by our search and, ultimately, ensure that the search did not miss any. This practice identified only one human observational study that observes the effects of heat exposure on pregnancy. The other human study, by Vaha-Eskeli, was included in Covidence as it observed placental outcomes. In 2022, Bonell et al. conducted an observational pilot study in West Kiang, The Gambia among 92 women who reported working in an outdoor setting (i.e., agriculture, water collection, gardening, etc.) [22]. Bonell et al. observed pregnant women every two months until delivery and assessed their fetal heart rate and fetal strain using ultrasound at the start and end of their outdoor work shift. The methods from this study are further outlined in Table 2 below.

The objectives of this systematic review are to analyze the available literature on the associations between heat stress exposure and placental function to synthesize methodologies and designs adopted in heat and placenta studies to inform approaches that should be implemented in future research examining heat and human placental function. This review will synthesize the results, considering methods, strengths, and limitations of the existing literature on heat stress and placental function in human and non-human studies. Specifically, the methods used within human and animal studies, including temperature exposure parameters (e.g., settings, temperature ranges, heating mode), pregnancy/gestation window of exposure, and placental outcome variables, will be discussed. While this review focuses on human methods and placental outcomes, most of the studies included are animal models. This is because most of the work on placentas and heat exposure has been conducted on animals, not humans. However, the current literature suggests that while they are of different species, animal and human placentas have paralleled anatomical and pathological similarities and that animal placentas play an important role in understanding human placentas [28,29,30].

This review is novel in its focus on placental function in both human and animal studies. Existing systematic reviews focus on a broad range of pregnancy outcomes but have not highlighted the importance of observing changes to the placenta. Additionally, it will compare the methodologies used in random controlled trials (RCTs) with those in epidemiological studies from other systematic reviews. The methodologies being employed in most of the current human studies will be discussed to better understand how heat stress is being measured and assessed in human pregnancy.

## 2. Materials and Methods

A protocol for this systematic review has been created and registered into the Open Science Framework (OSF) (pre-registration #463608). The Preferred Reporting Items for Systematic Reviews and Meta-Analysis (PRISMA) recommendations were used as a guide for this review [31].

### 2.1. Eligibility Criteria

We included empirical peer-reviewed articles that evaluated the association between heat stress and placental function in pregnant subjects. Eligibility criteria were the following for both animal and human studies: (i) published empirical research; (ii) peer-reviewed articles published in English or Spanish language; (iii) studies with measurement of placenta biology; (iv) studies with measurement of a heat source (heat intervention e.g., climate-controlled chambers, seasonal heat, or microwave device); and (v) animal or human studies. All studies included in our review had to meet all of the aforementioned inclusion criteria.

### 2.2. Information Sources and Search Strategy

A literature search was conducted to identify articles addressing the effects of heat stress on the placenta. The electronic databases PubMed, Cumulative Index to Nursing and Allied Health Literature (CINHAL), and Medline were utilized for the searches between April 2023 and March 2024. The assistance of a librarian was used to refine the search terms and strategy. The following keywords were searched using MESH terms: (“Pregnancy”[Mesh] OR pregnancy OR gestation) AND (“Heat Stress Disorders”[Mesh] OR heat exposure OR heat exhaustion OR heat stress) AND (“Placenta diseases”[Mesh] OR placenta OR uterine blood flow). Bibliographies from selected articles were appraised to ensure full study capture.

### 2.3. Study Selection Process

Our search resulted in 235 articles, which were imported into Covidence^®^ [32], a screening and data extraction tool, and were screened for duplication. After no duplicates were found, titles and abstracts were assessed by two independent reviewers to identify those that met the inclusion criteria. Twenty-four articles remained in the final sample for data extraction and summarization of results.

### 2.4. Extraction and Synthesis

Extraction of full text articles was performed in Covidence^®^ with summary data further extracted into a text file. A data extraction template was created, including title, author, year of publication, country in which the study was conducted, aims, study design, participants (number, description), temperature exposure parameters, pregnancy or gestation window of exposure, placental outcome variables, and main results. Due to the varying gestational ranges being investigated within the studies of different animal species (e.g., sheep, rats, buffalo, cows, goats, pigs, and humans) included in this review, exposure to environmental temperature was classified as either during various stages of pregnancy or during part of gestation, which we defined as early gestation, mid-gestation, and late gestation for each respective species (Table 3).

### 2.5. Assessment of Quality of Evidence

All studies in this review were thoroughly assessed for quality of evidence on Covidence based on sequence generation (the process of determining the order in which participants are allocated to different groups or conditions in a research study, particularly in randomized controlled trials (RCTs)), incomplete outcome data (the completeness of outcome data for each main outcome, including attrition and exclusions from the analysis), selective reporting (selective reporting of results found by reviewers), and other sources of bias (e.g., something pre-specified in the reviewers’ protocol not provided by authors). Two reviewers assessed all studies independently and then both reviewers discussed and came to a consensus on any differences. While most of the random control trials (RCTs) included were of high-quality evidence, three studies had selective outcome reporting of results [33,34,35]. For example, Adrianakis et al. reported speculation regarding an increase in uterine blood flow during maternal hyperthermia and how there might be an increase in blood flow in the uterus; however, this is contrary to the actual results of the study where placental blood flow did not increase [33]. Furthermore, two studies were at risk for bias [36,37].

## 3. Results

### 3.1. Literature Search

**Figure 1:**
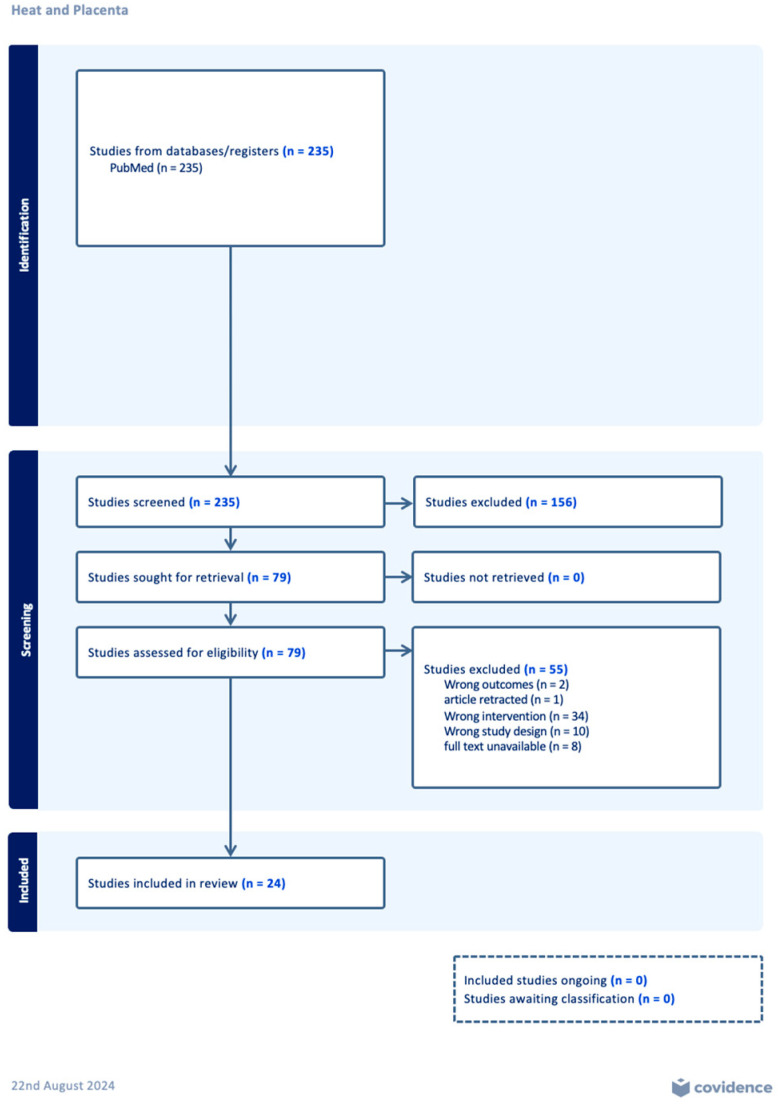
The PRISMA Flow Diagram illustrates the various stages of the methodology employed in this systematic review provided by the Covidence Systematic review software.

The database and citation search identified 235 potential articles for screening. After no duplicates were identified, title and abstract screening was performed on the 235 articles. A total of 79 full text articles were assessed for eligibility after 156 articles were excluded for being non-placental and non-empirical studies. The 79 articles that met inclusion, went for full text review to determine final eligibility. All conflicts were resolved through consultation between reviewers until a consensus was reached. After further excluding 55 articles (out of the 79) due to wrong intervention, full text availability, and wrong study design, a total of 24 articles proceeded to the data extraction phase. The remaining 24 articles met all inclusion criteria required for this systematic review (Figure 1).

### 3.2. Study Characteristics

Of the twenty-four articles in the final sample, eighteen were randomized controlled trials and six were non-randomized experimental studies. Studies included in this review were conducted between the years 1971 and 2023, with a little less than half of the articles taking place between 2020 and 2023 (*n* = 10). Several countries were represented in these studies, such as Australia (*n* = 6 Australian studies), the United States (*n* = 9 American studies), Brazil (*n* = 2 Brazilian studies), China (*n* = 2 Chinese studies) and Greece (*n* = 1 Greek study), Egypt (*n* = 1 Egyptian study), Finland (*n* = 1 Finnish study), Japan (*n* = 1 Japanese study), and United Arab Emirates (UAE) (*n* = 1 UAE study). Study samples included animal (*n* = 23 studies with animal subjects) and human (*n* = 1 study with human subjects) studies, with sample sizes ranging from 7 to 48 animal or human subjects. For animal studies, the species included consisted of sheep (*n* = 10 studies with sheep subjects), rats (*n* = 7 studies with rat subjects), buffalo (*n* = 1 study with buffalo subjects), cows (*n* = 1 study with cow subject), goats (*n* = 1 study with goat subjects), and pigs (*n* = 3 studies with pig subjects).

Fifteen studies used climate-controlled chambers or rooms with heat stress temperature conditions ranging from 28 to 48 °C. Of the remaining nine studies, four of them used seasonal heat exposure, two used a radiant fan heater or microwave exposure device, one used a sauna, and two studies did not specify the mode of heat exposure [38]. Sixteen of the twenty-four studies also measured relative humidity. Consistent with our inclusion criteria, all studies examined the effects of heat stress on placental function. Five of the twenty-four studies assessed the placenta during late gestation, eight during mid-gestation, two during early gestation, and nine during various stages of pregnancy. The methodologies used to assess the effects of heat exposure on placental outcomes consisted of (a) placental weight and efficiency (*n* = 14), (b) uteroplacental blood flow (*n* = 8), (c) placental anatomy (*n* = 7), (d) gene expression (*n* = 8), and placental steroids (*n* = 3). A summary of the studies presented in this review is found below in Table 4.

### 3.3. Findings of Heat Effects on the Placenta

#### 3.3.1. Weight and Efficiency

Placental weight and efficiency (the effectiveness of the placenta in supporting fetal growth) were the most used placental function measured in the studies included in this review (*n* = 14) [36,39,40,42,44,45,46,48,49,50,51,53,56,57]. Higher placental weights, lower placental efficiency, and lower fetal weights were observed in three studies that utilized a climate-controlled room or chamber with heat stress ranging from 28 to 44 °C [39,56,57]. Zhao et al. conducted two of these studies using cyclic heat stress to mimic hot Summer conditions (28–33 °C) for 3 weeks during early to mid-gestation in pregnant pigs [56,57] and, in the study by Arora et al., pregnant rats were exposed to heat stress (43–44 °C) for 90 min in the morning and 45 min in the afternoon on one day out of eight during various stages of pregnancy [39].

Conversely, lower placental weights were observed in eight studies [34,35,36,37,40,42,49,53] with two also reporting lower fetal weights [34,35] and two reporting no significant change in fetal weight [36,53]. All seven studies used climate-controlled rooms or chambers with heat stress conditions ranging from 29 to 40 °C. Galan et al. [34,35] experimented on pregnant ewes during mid-gestation (35–85 days gestation for the first group) and 35–115 days gestation for the second group and exposed their subjects to constant heat ranging from 35 to 40 °C throughout the day for around 7 weeks [34,35]. Bell studied ewes during mid and late gestation (Days 42–54 and 120 of gestation); the intervention group were placed in 38–40 °C temperatures for 9 h daily and then at 30–32 °C for the remaining 15 h with relative humidity at 40–50%. The study by Early et al. involved pregnant Suffolk ewes; those in the experimental group were in heat-stressed conditions of 30 °C with 40% humidity from 60 to 141 days of gestation [42]. Hensleigh and Johnson reported lower placental weight but no change to fetal weight when studying pregnant rats in a climate-controlled chamber with heat stress temperatures set at 39 °C for 4 h a day during early gestation [36]. Vatnick also reported lower placental weight and no change in fetal weight and subjected the experimental group to heat stress conditions of 40 °C for 12 h and 30 °C for the remainder of the day; relative humidity was not controlled but never over 50% [53]. The placentas of Wistar rats in the study by Padmanbhan weighed less when exposed to 42 °C than when exposed to 41 °C. Lastly, Olivier et al. [48] studied rats during late gestation with some rats exposed to heat stress at 29 °C and others going on to being exposed to up to 37 ºC for 8 h each day and then placed back in a 29 °C environment for the rest of the day. In the study by Regnault et al. [50], no change in placental weight was found after 56 days of heat exposure in a hyperthermic treatment chamber (40 °C for 12 h then 35 °C for 12 h) during mid-gestation (ewes; between Days 37 and 93 of pregnancy). Finally, in a study conducted by Silva et al. in Brazil analyzing 46 goats as their subjects during late gestation (last 60 days of pregnancy), lower placental efficiency was reported after heat exposure during the last 60 days of pregnancy [39]. Heat exposure was administered via a climatic chamber with a heating system that maintained the air temperature at 37 °C and the relative humidity at 60 to 70% from 08:00 to 16:00 h; after this period, the heating system was turned off and the air temperature and humidity returned gradually to the environmental conditions [39]. Placental weight and efficiency emerged as a frequently measured outcome in the studies reviewed. The observed variability in findings, with some studies reporting higher placental weights but lower efficiency and others demonstrating lower weights without consistent impacts on fetal weight, underscores the complexity of the relationship between heat stress and placental function. Notably, the diverse methodologies employed, including cyclic heat stress, seasonal exposure, and controlled chambers, contribute to the nuanced interpretation of results.

#### 3.3.2. Uteroplacental Blood Flow

Uteroplacental blood flow was assessed in eight studies [33,34,38,43,45,47,54,55]. Two of the studies used seasonal temperature measurements comparing the placental outcomes of animal models in the warm and cold seasons [43,47] and six studies used climate-controlled chambers or environments with cyclical heat stress ranging from 20 to 48 °C [33,34,38,43,45,54,55]. Uteroplacental blood flow was measured using various methods, ranging from more invasive (i.e., surgical) to non-invasive (i.e., ultrasonography). The most common method to observe the uteroplacental method was via B-mode and color ultrasonography 35 [48,50]. The second most common method was surgically implanting electromagnetic blood flow transducers near the mid-uterine artery or into the endometrium [34,38,54]. When using the electromagnetic blood flow transducers, uterine blood flow measurements were recorded immediately before heat stress [38] and in 15-min intervals following exposure [34]. Andrianakis et al. inserted catheters into the uterine and umbilical vessels and used the Fick indicator dilution method to obtain blood flow [33]. Uteroplacental blood flow, a crucial determinant of placental function, was a focus in eight studies. The diverse methods used for assessment, from ultrasonography to surgical interventions, highlight the multifaceted approaches to understanding vascular dynamics under heat stress conditions. Findings indicate that heat stress can indeed influence uteroplacental blood flow, with potential consequences for fetal development.

#### 3.3.3. Placental Anatomy

Seven studies analyzed placental anatomy and almost all found significant changes when exposed to heat stress [41,48,49,54,55,56,57]. The outcomes for placental anatomy included cotyledon count, placental mass, placental surface area, uteroplacental thickness, middle uterine artery diameter, placental structure, and placental histology [41,48,49,54,55,56,57]. Six studies [48,49,54,55,56,57] used climate-controlled cabinets or facilities and four [48,54,56,57] out of these six studies used cyclic heat exposure (~8 h of daily heat exposure) while one [49] exposed the subject to 1 h of intense heat exposure (41–42 °C). Another study used seasonal heat (Summer season) [43] and one compared an open pastoral (OP) system (no trees) to a silvopastoral (SP) system (trees), looking at animals in different controlled spaces with and without tree cover [41]. Temperatures in studies focusing on placental anatomy ranged between 26 and 43 °C. The placental surface area was increased with maternal heat treatment [57]. Most studies reported placental morphological and structural changes with heat intervention except for Dada et al., who did not detect a system effect between OP and SP systems regarding the total area of cotyledons per placenta, the area or the mass of the placental membrane [41]. The consistent identification of significant alterations, such as changes in cotyledon count, placental mass, and surface area, suggest a robust relationship between heat stress and structural modifications in the placenta. However, the varying methodologies, including cyclic heat exposure and seasonal variations, underscore the need for further exploration into the specific mechanisms driving these changes.

#### 3.3.4. Gene Expression

Gene expression was measured in eight studies in this review [36,37,42,48,50,51,56,57]. All studies measuring gene expression used climate-controlled chambers to simulate cyclic heat stress (ranging between 8 and 12 h intervals) with intervention heat temperatures ranging from 28 to 40 °C. The study by Early indicated that chronic heat stress in pregnant ewes reduced the overall capacity for protein synthesis in the placenta, as evidenced by decreased total RNA and protein content, as well as lower RNA-to-DNA ratios [42]. Overall, chronic heat exposure was shown to lower circulating placental hormone concentrations, affecting gene expression related to fetal development, and alter placental mRNA expression, indicating a range of impacts on the maternal–fetal interface.

#### 3.3.5. Placental Steroids

Placental steroids were measured in three studies [43,51,52]. Studies were conducted on buffalo, sheep, and humans. The one human study by Vaha-Eskeli et al. investigated the effects of heat stress on various biochemical markers in pregnant and non-pregnant women. The participants were divided into three groups: healthy non-pregnant women, women who were 13–14 weeks pregnant, and women who were 36–37 weeks pregnant. During the study, blood samples were collected from the participants before, during, and after exposure to heat stress in a sauna (70 °C for 20 min with 15% relative humidity followed by a 45 min recovery period at a room temperature of 21–23 °C). The researchers measured levels of plasma prostacyclin (stimulates vasodilation) and thromboxane A2 (stimulates activation of new platelets), as well as the placental steroids serum estradiol, estriol, and progesterone. Rectal and skin temperatures were recorded with an electric thermometer throughout heat exposure [52]. The concentration of the placental steroids was measured by radioimmunoassay [52]. The results of this study showed that progesterone levels remained stable throughout the experiment, estradiol levels increased significantly in both pregnant groups after exposure to heat stress, and estriol levels and the metabolite of prostacyclin increased only in the group of women who were 36–37 weeks pregnant. The metabolite of thromboxane A2 decreased in the group of women who were 13–14 weeks pregnant at the end of the stress. Despite these changes, the study found that fetal well-being, as indicated by fetal heart rate reactivity and uterine contractions, remained unchanged. The researchers concluded that the small changes observed in the levels of these biochemical markers do not seem to have any harmful effects on fetal health. They suggest that the slight increase in placental steroids may reflect changes in metabolism rather than an increase in uteroplacental blood flow [52]. In the study by Silva, the increased expression of MC2R and NR3C1 genes in heat-stressed goats suggests an activation of the glucocorticoid pathway, likely leading to higher cortisol levels. The increased expression of HSPA1A indicates a physiological response to heat stress, reflecting cellular stress and protective mechanisms [51]. Lastly, in the study by El-Sherbiny, the results indicate that L-Arg administration positively affects placental steroid production in mid-pregnant buffalo cows exposed to heat stress, supporting the endocrine function of the placenta, potentially improving the hormonal environment necessary for fetal development [43]. These results prompt consideration of the experimental design’s representativeness, cross-species variations, and the clinical implications of understanding placental steroid dynamics during pregnancy.

## 4. Discussion

### 4.1. Literature Search and Study Characteristics

The comprehensive literature search yielded 22 articles that met the inclusion criteria, representing a diverse range of study designs and interventions. The majority of studies were randomized controlled trials (RCTs), reflecting the rigor often associated with experimental research. The inclusion of non-randomized experimental studies and an observational cohort study enhances the diversity of evidence considered in this review.

The temporal distribution of studies across the years highlights the enduring interest in understanding the impact of heat stress on placental function. While the studies span from 1971 to 2023, the concentration of research around 2020–2023 may reflect an increased awareness of environmental factors influencing pregnancy outcomes during this period. The global representation of study locations underscores the widespread concern regarding the effects of heat stress on placental health and pregnancy. The inclusion of both animal and human subjects in the reviewed studies adds complexity to our understanding, recognizing the translational relevance of animal models to human physiology.

### 4.2. Study Methodology and Design Implications to Heat and Placenta Studies with Human Subjects

In human studies, the methodologies relied on gestational age (early vs. late pregnancy), whereas, with animals, the methodologies relied on temperature control at several time points in pregnancy. Of the twenty-two studies in this review, only one was conducted with human subjects [35]. The study conducted in Finland aimed to study the hormonal responses to heat stress during early and late pregnancy and the non-pregnant state in an attempt to find out whether the hormonal responsiveness is altered by pregnancy and whether fetal well-being could be compromised in utero. This study utilized three groups of pregnant women. Group 1 or the control group, was comprised of 15 healthy non-pregnant women. Group 2, comprised 23 women in mid gestation at 13–14 weeks pregnant. Group 3 comprised women (*n* = 23 women) in late gestation at 36–37 weeks pregnant. The experiment started off with a 20-min resting period at room temperature, followed by 20 min of thermal stress at 70 °C for 20 min with 15% relative humidity followed by a 45 min recovery period at a room temperature of 21–23 °C. Rectal and skin temperatures were recorded with an electric thermometer. Uterine contractility and fetal heart rate were recorded using an external cardiotocograph and the concentration of placental steroids was measured by radioimmunoassay. The major design implication of this is that we cannot replicate the intervention by Vaha-Eskeli because of the health ethical risks of exposing pregnant women to thermal stress. Future work should be observational regarding temperature.

Of the animal studies, the most common study designs were RCTs that used climate-controlled chambers or rooms with heat stress in an open-pasture or silvopasture setting. The one human study that met the criteria for this review also used a climate-controlled exposure (e.g., sauna) and observed changes to hormonal responsiveness in an early- and late-pregnancy heat-stressed placenta [35]. The animal studies and human study both utilized a fixed temperature exposure period at various time points in pregnancy. Other human studies used an observational approach that observes changes in pregnancy outcomes based on seasonal heat changes [22]. These studies did not meet the criteria for this review due to a lack of assessing placental outcomes. The current literature with pregnant humans and heat exposure is primarily observational since it is difficult to exposure humans to certain temperature conditions. While there is extensive literature involving heat and pregnancy outcomes, the outcomes related specifically to the placenta remain understudies. This gap in research should guide future work to focus on observing the effects of temperature and humidity on genetic changes to the placenta.

## 5. Conclusions

The collective evidence presented in this review highlights the multifaceted impact of heat stress on placental function, encompassing changes in weight, efficiency, blood flow, anatomy, and gene expression. There is a need for more human placental studies to inform research on anatomical and physiological placental changes during heat exposure. Understanding these diverse effects is critical for informing clinical practice and public health interventions, particularly in the context of rising global temperatures.

Future research should aim to elucidate the specific mechanisms underlying the observed changes, considering the varied methodologies and species-specific responses. Additionally, investigations into potential mitigating factors, such as adaptation mechanisms or therapeutic interventions, may contribute to the development of strategies to safeguard placental and pregnancy health in the face of environmental challenges.

In conclusion, this review provides a comprehensive synthesis of the existing literature on the effects of heat stress on placental function, shedding light on the intricate interplay between environmental factors and pregnancy outcomes. The findings underscore the need for continued research to inform evidence-based strategies for maternal and fetal health in a changing climate.

## Figures and Tables

**Table 1:** Summary of methodologies of human study designs, temperature measurement methods, and windows of exposure of other reviews.

Authors	Total Studies Included	Methodologies Reviewed	Study Designs	Temperature Parameters	Methods for Temperature Measurements	Pregnancy Window Exposure	Pregnancy Outcome Variables
Beltran et al., 2014 [25]	90	No	N/A	Tropical versus non-tropical	Min and max daily temperatures, precipitation, relative humidity	First, second, and third trimesters Last month of pregnancy	Hypertensive disorders of pregnancy, length of gestation, birth weight
Bekkar et al., 2020 [26]	68	Yes	Forty-nine cohort studies Six case-crossover Five cross-sectional Four time series Four case-control	Seasonal Monthly Weekly	Weekly mean apparent temperature, weekly mean extreme temperature	Second and third trimesters Week before delivery	Preterm birth Low birth weight Stillbirth
Chersich et al., 2020 [17]	70	No	N/A	Heat wave and non-heatwave days	OR and hazard ratios of preterm birth, low birth weight, and stillbirth on heat wave days	Exposure during one trimester or all trimesters Exposure period <4 weeks	Preterm birth Birthweight Stillbirth
Strand et al., 2011 [27]	32	No	N/A	Seasonal heat patterns	Mean weekly temperatures and humidity index Daily min and max	Each trimester Middle 10 days of each trimester A total of 10 days around conception Day of birth Month of birth	Preterm birth Stillbirth Low birth weight
Syed et al., 2022 [14]	83	Yes	Thirty-one cohort Sixteen population registry Thirteen time registry Eleven case-crossover Seven case-control Two case series One not stated	Hourly Daily Monthly Seasonal Climate Heatwave	Temperature data obtained from regional monitoring stations	First trimester Second and third trimesters Third trimester only All/any part of the pregnancy Delivery date	Preterm birth Birthweight Congenital anomaly Stillbirth

**Table 2:** Human observational study.

Author	Study Design	Population	Data Collection Timeframe	Temperature Exposure Parameters	Pregnancy Window of Assessment	Methods for Temperature Assessment	Pregnancy Outcome Variables	Significant Findings
Bonell et al., 2022 [22]	Observational cohort	*n* = 92 Inclusion criteria: those who were 16 years or older and at <36 weeks’ gestation and involved in agricultural or related manual daily tasks of living Exclusion criteria: those who refused to consent, had multiple pregnancies, were acutely unwell, or were diagnosed with pre-eclampsia or eclampsia	2019–2020	Rainy season (August–October) First half of the dry season (November–March)	Every 2 months during pregnancy until delivery	Heat stress was measured during work shifts by wet bulb globe temperature (WBGT) and by using the universal thermal climate index (UTCI). Maternal heat strain was measured by the modified physiological strain index calculated from heart rate (via ultrasound) and skin temperature	Fetal heart rate, fetal strain	Significant increase in FHR from baseline at rest (125 [SD 7.9] bpm) to the working period (147 [SD 11.9] bpm; *p* < 0.0001 was found. The total effect of UTCI on fetal strain resulted in an odds ratio (OR) of 1.17 (95% CI 1.09–1.29; *p* < 0.0001), with an adjusted direct effect of OR of 1.12 (1.03–1.21; *p* = 0.010).

**Table 3:** Gestational periods for animals and humans.

Sheep	Rats	Buffalo	Cows	Goats	Pigs	Human
Early: Day 1–50	Early: Day 1–7	Early: Day 1–100	Early: Day 1–95	Early: Day 1–50	Early: Day 1–38	Early: conception to 12 weeks
Mid: Day 51–100	Mid: Day 8–15	Mid: Day 101–200	Mid: Day 96–191	Mid: Day 51–100	Mid: Day 39–77	Mid: 13–27 weeks
Late: Day 101–150±	Late: Day 16–23±	Late: Day 201–300±	Late: Day 192–285±	Late: Day 101–150±	Late: Day 78–115±	Late: 28–40 weeks ±
Total average Gestation in Days: 150–153	Total average Gestation in Days: 21–24	Total average Gestation in Days: 305–320	Total average Gestation in Days: 279–289	Total average Gestation in Days: 145–155	Total average Gestation in Days: 115	Total average Gestation in Weeks: about 40 weeks

**Table 4:** Summary of studies presented in the review.

Authors/Title/Country/ RCT or NON-RCT	Population	Pregnancy Window of Exposure	Temperature Exposure Parameters	Placental Outcome Variables	Major Results	Risk of Bias (Rated by Two Independent Reviewers)
Andrianakis and Walker, 1994 [33] Effect of Hyperthermia on Uterine and Umbilical Blood Flows in Pregnant Sheep Australia NON-RCT	Nine pregnant Border Leicester-Merino cross-bred sheep.	Total of 125–142 days (late) gestation.	Three radiant fan heaters were used to expose subjects to 43 °C for 8 h. The normal air-conditioned temperature was 21 °C with 30% relative humidity.	Uteroplacental blood flow.	The fetal temperature increased by 1.39 ± 0.12 °C, more than the maternal temperature increase of 1.19 ± 0.15 °C. there was a significant rise in uterine blood flow during hyperthermia (*p* = 0.006). Umbilical blood flow did not change significantly, but minor changes correlated with decreased fetal arterial Pco2.	Sequence generation: High. Incomplete outcome data: High. Selective reporting: Low (failed to report all results).
Arora et al., 1979 [39] Fetal and Placental Responses to Artificially Induced Hyperthermia in Rats United States RCT	Eight groups of six to eight outbred nulliparous Spartan female rats weighing 250–275 g.	Various stages of pregnancy.	Pregnant rats were heat-stressed on Gestation Days 4, 6, 8, 10, 12, 14, 16, or 18 for 90 min in the morning and 45 min in the afternoon at 43–44 °C in an incubator chamber.	Placental weight	Heat treatment significantly suppressed maternal weight gain and reduced embryo survival when applied on Day 4 of pregnancy. Placentas from Day 6–10 treatment groups were significantly heavier than controls, showing extensive thickening and necrosis of decidua basalis. Although the impact on fetal circulation is unclear, most fetuses associated with heat group placentas were smaller than controls.	Sequence generation: High. Incomplete outcome data: High. Selective reporting: High.
Bell et al., 1987 [40] Some aspects of placental function in chronically heat-stressed ewes United States Non-RCT	Group 1: experimental group (heat stress) of five single-pregnant Columbia-Rambouillet crossbred ewes. Group 2: five single pregnant ewes from the same stock not exposed to heat.	Between Days 42–54 and 120 of gestation (mid and late)	In the climatic chamber, the dry bulb air temperature was maintained at 38–40 °C for 9 h daily and 30–32 °C for the remaining 15 h, with relative humidity at 40–50%.	Uteroplacental blood flow, placental weight.	Heat exposure significantly reduced placental weight, uterine and umbilical blood flows, and placental glucose transfer capacity. These reductions were highly correlated with placental weight.	Sequence generation: High. Incomplete outcome data: High. Selective reporting: High.
Dada et al., 2023 [41] Placental Development and Physiological Changes in Pregnant Ewes in Silvopastoral (SP) and Open Pasture (OP) Systems during the Summer Brazil RCT	Twenty-four pregnant Dorper × Santa Inês crossbred ewes. They were divided into groups of 12 animals each and subjected to two treatments according to the rearing systems: the silvopastoral (SP) system and the open pasture (OP) system.	Third trimester 50 days gestation to 123.4 days gestation (mid and late).	In the OP, females remained in the open air without shade, while in the SP, they had tree shade. Both systems were stressful but the SP system had a lower air temperature than the OP system (26.0 ± 0.38 °C vs. 26.9 ± 0.41 °C; *p* = 0.0288). The microclimate of both environments was evaluated by studying air temperature (AT, °C), black globe temperature (BGT, °C), dew point temperature (DPT, °C), wind speed (WS, m/s), and relative humidity (RH, %).	Placental anatomy (placental mass, placental area, and the number of cotyledons were counted).	There was no system effect (OP vs. SP) on the total area of cotyledons per placenta, the area, and the mass of the chorionic membrane (*p* = 0.8456, *p* = 0.7178, and *p* = 0.9092, respectively).	Sequence generation: High. Incomplete outcome data: High. Selective reporting: High.
Dreiling et al., 1991 [38] Maternal Endocrine and Fetal Metabolic Responses to Heat Stress United States RCT	Pregnant ewes of mixed breeding.	Total of 110–130 days gestation (mid gestation).	Temperature of 24 °C for 40 min then 48 °C for 75 min daily.	Uteroplacental blood flow.	After 40 min, UBF dropped to 70% of the control rate. Fetuses from heat-stressed ewes weighed 82% of those from normal conditions. Heat-induced ADH or OT secretion may significantly impact uteroplacental perfusion in near-term fetuses.	Sequence generation: High. Incomplete outcome data: High. Selective reporting: High.
Early et al., 1991 [42] Chronic heat stress and prenatal development in sheep: II. Placental cellularity and metabolism United States RCT	A total of 13 pregnant Suffolk ewes. Ewes were then assigned by BW and litter size to either a thermoneutra18 (TN) or heat9 (H) treatment beginning on Day 64 of gestation.	Total of 60–141 days gestation (mid and late gestation).	Pregnant ewes subjected to either thermoneutral (18 to 20 °C, 30% humidity, *n* = 7) or hot (H, 30 to WC, 40% humidity, *n* = 5) temperatures.	Placental weight and gene expression	Fetal and placental weights, as well as protein, RNA, and DNA content, were significantly reduced (*p* < 0.001) in heat-stressed (H) ewes. Heat stress notably decreased total cell number and placentae size, with only a slight reduction in cell size. Maternal placenta and myoendometrium oxygen consumption remained unchanged.	Sequence generation: High. Incomplete outcome data: High. Selective reporting: High.
El-Sherbiny et al., 2022 [43] Exogenous L-arginine administration improves uterine vascular perfusion, uteroplacental thickness, steroid concentrations, and nitric oxide levels in pregnant buffaloes under subtropical conditions Egypt RCT	Pluri-parous (*n* = 12) pregnant buffaloes.	Mid-gestation: ~180–190 days.	In August 2021, during mid-Summer, the Temperature Humidity Index (THI) exceeded 85, indicating severe heat stress for the animals. Twelve buffalo were randomly assigned to two groups: one group received a 5 mg/kg BW intravenous bolus of L-arginine (L-Arg) (*n* = 6; ARG), a nitric oxide (NO) precursor, while the control group (*n* = 6; CON) received 25 mL of normal saline. Measurements were taken at various time points: −1, 0, 2, 4, 24, 48, 72, 96, and 120 h post-L-Arg administration.	Placental steroids and uteroplacental blood flow.	L-Arg improved uteroplacental thickness and uterine hemodynamics in heat-stressed buffalo cows. Combined uteroplacental thickness (CTUP) was higher (*p* < 0.05) in the ARG group at H96 to 120 and L-Arg enhanced NO levels and steroid production. Measurements of CTUP, MUA diameter, and Doppler hemodynamics were taken with B-mode and color Doppler ultrasonography.	Sequence generation: High. Incomplete outcome data: High. Selective reporting: High.
Galan et al., 1999 [44] Relationship of fetal growth to duration of heat stress in an ovine model of placental insufficiency United States RCT	Ten pregnant ewes—five ewes were housed in an environmental chamber beginning at 35 days’ gestation and were removed after 55 days of exposure (Heat 55). Five ewes served as a control group and were housed at 20 °C ambient temperature.	A total of 35–85 days gestation for the first group (Heat 55) and 35–115 days gestation for the second group (heat 80) (mid).	Temperature maintained at 40 °C for 18 h during the day and decreased to 35 °C at night and humidity kept between 35% and 40%.	Placental weight.	Both heat groups had significantly lower fetal and placental weights compared with those in the control group. The Heat 80 group had significantly lower fetal and placental weights compared with those of the Heat 55 group.	Sequence generation: High. Incomplete outcome data: High. Selective reporting: High.
Galan et al., 1998 [45] Doppler velocimetry of growth-restricted fetuses in an ovine model of placental insufficiency United States RCT	Ten mixed-breed (Columbia-Rambouillet) ewes with singleton pregnancies were used for this study. Five ewes were housed in an environmental chamber for 55 days beginning at 35 days gestation and five ewes that served as controls were housed at 20 °C ambient temperature.	A total of 35–85 days gestation (mid-gestation).	Temperature maintained at 40 °C for 12 h during the day and decreased to 35 °C at night and humidity kept between 35% and 40%, for 55 days.	Placental weight and efficiency.	Heat-stressed fetuses had higher systolic/diastolic ratios and pulsatility index values in the umbilical artery and aorta (*p* < 0.033). Their placentas weighed less (181.9 ± 7.0 gm vs. 307.06 ± 22.2 gm; *p* = 0.002). These differences appeared after 80 days of gestation.	Sequence generation: High. Incomplete outcome data: High. Selective reporting: High.
Guo et al., 2022 [37] Heat Stress Modulates a Placental Immune Response Associated with Alterations in the Development of the Fetal Intestine and Its Innate Immune System in Late Pregnant Mouse China RCT	ICR strains of female and male mice.	Late gestation.	The control (TN) group mice were kept at room temperature (24 ± 1 °C) and the HS group mice were kept in the artificial intelligence climate chamber (35 ± 1 °C).	Gene expression.	Data showed that maternal heat stress reduced the expression of genes linked to fetal intestinal development, potentially due to increased expression of cell cycle genes. It also inhibited genes related to the fetal intestinal innate immune system, possibly due to placental inflammatory responses.	Sequence generation: Low. Did not specify how control and intervention groups were comparable. Incomplete outcome data: High. Selective reporting: High.
Guo et al., 2021 [36] Heat stress affects fetal brain and intestinal function associated with the alterations of the placental barrier in late pregnant mouse China RCT	Nineteen ICR strain mice.	E12 to E18 (mid to late gestation).	Parameters of 35 °C, 70% humidity artificial intelligence climate chamber (35 ± 1 °C).	Placental efficiency and gene expression.	Maternal heat stress harmed placental development, increased fetal deaths, and disrupted lipid metabolism. It up-regulated nutrient transport genes in the placenta but down-regulated them in the fetal intestine. TG levels rose in the placenta and fell in the fetal intestine, affecting fetal brain function and the serotonin system.	Sequence generation: Low. Did not specify how control and intervention groups were comparable. Incomplete outcome data: High. Selective reporting: High.
Hensleigh and Johnson, 1971 [46] Heat Stress Effects During Pregnancy. I. Retardnation of Fetal Rat Growth United States RCT	Pregnant rats of the Holtzman strain were obtained on or before Day 5 of gestation.	Early stages of pregnancy (Days 7–11).	High ambient temperature (39 °C; 58% relative humidity) for 4 h/day on Days 7–11. Kept in the chamber where temperatures were controlled.	Placental Weight	Heat exposure reduced fetal and placental weight on Day 12. On Day 20, only fetal weight was reduced, even without stress after Day 11. Ovariectomized rats on estrone and progesterone had normal fetuses and larger placentas compared to controls. Heat stress caused a significant reduction in placental weight on Day 20 but fetal size remained normal.	Sequence generation: Low. Did not specify how control and intervention groups were comparable. Incomplete outcome data: High. Selective reporting: High.
Nakamura et al., 2000 [34] Heat Produces Uteroplacental Circulatory Disturbance in Pregnant Rats through Action of Corticotropin-Releasing Hormone (CRH) Japan RCT	A total of 24 virgin female Wistar rats and 24 Wistar rats at 15–16 days gestation were studied.	Mid pregnancy	The environment was maintained at 21–23 °C with 50–60% humidity, with 12-h light (08:00–20:00) and 12-h dark cycles. Microwaves at 2 mW/cm^2^ power density and 2450 MHz were applied for 90 min using a microwave exposure device.	Uteroplacental blood flow was monitored by the hydrogen gas electrolytic method.	Higher uteroplacental blood flow in pregnant rats than uterine blood flow in virgin rats. Heat and α-helical CRH significantly affected uteroplacental blood flow in pregnant rats but not in virgin rats. Blood flow decreased significantly from 15 to 90 min in pregnant rats without α-helical CRH, with no change in those with α-helical CRH.	Sequence generation: High. Incomplete outcome data: High. Selective reporting: Low (failed to report all results).
Nanas et al., 2021 [47] Ultrasonographic findings of the corpus luteum and the gravid uterus during heat stress in dairy cattle Greece NON-RCT	A total of 19 pregnant dairy cows (Winter group (*n* = 9) and Summer group (*n* = 10)).	Mid-pregnancy 90–110 days.	During the cool period of October to April (examined in March) and warm period of May to September (examined mid-August), the farm was equipped with an electronically operating cooling system and automatic activation of fans and sprinklers. Temperature humidity index (THI) was also measured.	Uteroplacental blood flow (the umbilical and uterine artery diameters and hemodynamic parameters in the vessels were calculated using the pulse wave doppler).	Seasonal variations did not affect hemodynamic parameters, uterine artery diameter, or placentome length. The grey-scale intensity and low color flow in placentomes were higher in Winter. Group S had lower umbilical artery diameter and blood volume than group W. Uterine artery blood flow was unaffected. In humans, brief late-pregnancy heat stress increases heart rate but not uterine vascular resistance.	Sequence generation: High. Incomplete outcome data: High. Selective reporting: High.
Olivier et al., 2021 [48] Maternal, Placental, and Fetal Responses to Intermittent Heat Exposure During Late Gestation in Mice Australia RCT	21 C57BL/6J mice	Fetal and placental tissues were collected at E18.5 (late gestation).	Three groups were studied: 1. SH Group (*n* = 7) Housed at 22 °C from E0.5 to E18.5; 2. TNZ Group (*n* = 7): Housed at 29 °C (thermoneutral zone) from E0.5 to E18.5; 3. HW Group (*n* = 7): Housed at 29 °C until E15.5, then placed in a 37 °C incubator from 09:00 to 17:00 on E15.5, E16.5, and E17.5, and returned to 29 °C for the rest of the time. Temperatures were 29.2 ± 0.5 °C in the warming cabinet and 37.7 ± 0.5 °C in the incubator.	Placental weight and efficiency; gene expression; and placental anatomy.	Housing temperature significantly affected placental weight, which was 16% lower in the heat exposure group than in the TNZ group due to a smaller labyrinth zone. Vegfa expression was 37% lower in the heat exposure group compared to the SH group. The expressions of Hsd11b2, Slc2a3, Slc38a2, and Slc38a4 were unchanged. In the fetal brain, Hif1α expression was 45% lower in the heat exposure group than in the TNZ group (*p* < 0.05).	Sequence generation: High. Incomplete outcome data: High. Selective reporting: High.
Padmanabhan et al., 2005 [49] Histological, histochemical and electron microscopic changes of the placenta induced by maternal exposure to hyperthermia in the rat United Arab Emirates RCT	Wistar rats *n* = 34: *n* = 14 at 41 °C, *n* = 10 at 42 °C, and *n* = 10 controls.	GD9–GD20—when embryos were at the beginning of organogenesis (mid and late Gestation).	Temperature of 41 °C for one group and 42 for the other for one hour on GD9 polypropylene cage and put in an incubator chamber pre-heated to 41 °C (*n* = ¼ 14) or 42 °C (*n* = ¼ 10) for 1 h.	Placental weight and placental anatomy.	Most placentas in the hyperthermia groups (82%) had a white fibrinoid patch at the margin. Only the 42 °C group’s placentas were lighter than the controls. Heat-stressed embryos had thicker decidua basalis, with more pronounced glycogen cell degeneration in the 42 °C group than the 41 °C group, leading to cyst formation in the basal zone.	Sequence generation: High. Incomplete outcome data: High. Selective reporting: High.
Regnault et al., 1999 [50] Altered arterial concentrations of placental hormones during maximal placental growth in a model of placental insufficiency United States NON-RCT	A total of 14 pregnant 2–3-year-old Columbian Rambouillet ewes with a single fetus.	Between Days 37 and 93 of pregnancy (mid-gestation).	Temperature of 40 °C for 12 h then 35 °C for 12 h, 30–40% relative humidity. Hyperthermic treatment chamber.	Placental weight and efficiency and gene expression.	Chronic heat stress reduced progesterone and ovine placental lactogen (oPL) levels in ewes but did not significantly affect oPL mRNA or protein in cotyledonary tissue compared to TN ewes. Prolactin levels were four times higher in heat-stressed ewes (*p* < 0.0001) while placental weights were not significantly affected.	Sequence generation: High. Incomplete outcome data: High. Selective reporting: High.
Silva et al., 2020 [51] Heat stress affects the expression of key genes in the placenta, placental characteristics, and efficiency of Saanen goats and the survival and growth of their kids Brazil RCT	A total of 46 Saanen goats were used: 14 primiparous and 32 multiparous.	The last 60 days of pregnancy until the first colostrum suckling.	The subtropical area has a 23 °C average temperature, 73% humidity, a rainy season from November to March, and 1300–2000 mm annual rainfall. Data were collected between June and September. Goats were in a control group (CT; *n* = 23) or heat-stressed group (HS; *n* = 23) in a chamber at 37 °C and 60–70% humidity from 08:00 to 16:00 h, with conditions returning to normal after that.	Placental weight and efficiency; placental steroids; and placental gene expression.	Heat-stressed (HS) goats had longer placenta delivery times, more cotyledons, lighter cotyledons, and lower placental efficiency compared to control (CT) goats. HS goats showed higher MC2R and NR3C1 gene expression, lower HSD11B2 expression, and higher HSPA1A expression. There were no effects on NR3C2, HSP1, HSPD1, or HSPB1 gene expression and HSD11B1, CRH, and CRHR1 were not expressed in either group.	Sequence generation: High. Incomplete outcome data: High. Selective reporting: High.
Vaha-Eskeli et al., 1992 [52] Responses of placental steroids, prostacyclin, and thromboxane A2 to thermal stress during pregnancy Finland NON-RCT	Group 1: *n* = 15 healthy non-pregnant women; Group 2: *n* = 23 women 13–14 weeks pregnant; Group 3: *n* = 23 women 36–37 weeks pregnant; *n* = 47.	Early gestation (Group 2) and late gestation (Group 3).	The sauna protocol involved heating at 70 °C for 20 min with 15% humidity, followed by a 45-min recovery period at 21–23 °C. Rectal and skin temperatures were measured using an electric thermometer (TE3, Electrolaboratoriet Ellab A/S, Denmark).	Placental Steroids	The small changes in proteinoids and placental steroids due to heat stress appear not to negatively affect fetal well-being. The slight increase in placental steroids likely reflects metabolic changes rather than enhanced uteroplacental blood flow. In group II, E levels peaked during stress (15–40 min, 11%, *p* < 0.005) and remained elevated during recovery (*p* < 0.01).	Sequence generation: High. Incomplete outcome data: High. Selective reporting: Low (failed to report all results).
Vatnick et al., 1991 [53] Effect of Heat Stress on ovine placental growth in early pregnancy United States RCT	A total of 11 Ditocous Dorset ewes.	Between days 50 and 75 of gestation.	Temperature of 40 °C dry bulb from 8:00 to 17:00 h and 30 °C dry bulb for the remainder of the day. Relative humidity was not controlled in either room but never exceeded 50% during the entire experiment.	Placental weight.	Heat reduced placental weight by 19% but did not affect fetal weight. Placental DNA and protein concentrations, as well as protein/DNA ratios, were similar between groups. Total Placental DNA content was lower in heated ewes, indicating fewer cells, although the DNA synthesis rate tended to be higher.	Sequence generation: High. Incomplete outcome data: High. Selective reporting: High.
Walker et al., 1995 [54] Cardiovascular responses to heat stress in late gestation fetal sheep Australia NON-RCT	Seven pregnant ewes.	A total of 130–136 days gestation (late gestation).	To assess the impact of raising environmental temperature from 20 °C to 43 °C for 8 h at 30–60% relative humidity on uteroplacental blood flow and cardiac output distribution, 15 µm diameter radioactive microspheres were used in temperature-controlled rooms.	Uteroplacental blood flow and placental anatomy (the counts for all cotyledons were subsequently combined to determine the maternally derived placental blood flow).	Heat stress did not affect blood flow to the fetal or maternal sides of the placenta, indicating that perfusion-dependent heat transfer from fetus to mother does not increase under hyperthermic conditions.	Sequence generation: High. Incomplete outcome data: High. Selective reporting: High.
Zhao et al., 2022 [55] Heat stress of gilts around farrowing causes oxygen insufficiency in the umbilical cord and reduces piglet survival Australia RCT	Sixteen pregnant primiparous sows (gilts).	A total of 110 days—farrowing completion (late gestation).	Pregnant gilts were assigned to either a thermoneutral control (CON, *n* = 8; constant 20 °C) or cyclical heat stress (HS) condition (30 °C from 09:00 to 17:00 h and 28 °C from 17:00 to 09:00 h) in a climate-controlled facility at the University of Melbourne.	Uteroplacental blood flow and placental anatomy.	Heat-stressed gilts took longer to expel placentas (*p* = 0.003) and had higher stillbirth rates (*p* < 0.001). Surviving piglets had lower umbilical vein oxygen levels (*p* = 0.04) and oxygen saturation (*p* = 0.03), with higher lactate levels (*p* = 0.07). No retained piglets or placentas were observed in controls.	Sequence generation: High. Incomplete outcome data: High. Selective reporting: High.
Zhao et al., 2020 [56] Controlled elevated temperatures during early-mid gestation cause placental insufficiency and implications for fetal growth in pregnant pigs Australia RCT	*n* = 15 pregnant pigs; *n* = 7 thermoneutral; and *n* = 8 cyclic elevated temperature conditions.	Mid-gestation: d40–d60.	Pigs were housed under constant thermoneutral (*n* = 7) or cyclic elevated temperatures (33 °C from 09:00 to 17:00 h and 28 °C from 17:00 to 09:00 h; *n* = 8). Thermal stress was assessed weekly by measuring skin temperature and respiration rate at multiple times.	Placental weight and efficiency; placental anatomy; and gene expression.	ET fetuses had larger placentas (*p* = 0.041) but lower placental efficiency (*p* = 0.013). They showed reduced muscle fiber density (*p* = 0.032) and a thicker placental epithelial layer (*p* = 0.017). ET decreased GLUT-3 mRNA (*p* = 0.026) and increased IGF-2 mRNA (*p* = 0.037). Plasma progesterone was lower in ET pigs (*p* = 0.050), but progesterone per unit placental mass was unaffected.	Sequence generation: High. Incomplete outcome data: High. Selective reporting: High.
Zhao et al., 2021 [57] Maternal Heat Stress Alters Expression of Genes Associated with Nutrient Transport Activity and Metabolism in Female Placentae from Mid-Gestating Pigs Australia RCT	Ten large White × Landrace female pigs were selected from a commercial piggery and artificially inseminated on Day 0 at a farm.	A total of 40–60 days gestation (early to mid-gestation).	On Day 40 of gestation, pregnant pigs were exposed to either constant thermoneutral conditions (CON; *n* = 5; 20 °C) or cyclic heat stress (HS; *n* = 5; 33 °C from 09:00 to 17:00 h and 28 °C from 17:00 to 09:00 h) for 3 weeks in a climate-controlled facility, simulating Australian Summer conditions.	Placental weight and efficiency; placental anatomy; and placental gene expression.	On Day 60 of gestation, maternal heat treatment tended to decrease fetal weight (*p* = 0.08) but increase placental weight (*p* = 0.053) and surface area (*p* = 0.09). Placental efficiency was lower in the heat-stressed group (*p* = 0.023). Heat stress upregulated 35 genes and downregulated 134 genes, particularly those related to transporter and catalytic activities.	Sequence generation: High. Incomplete outcome data: High. Selective reporting: High.

## Data Availability

No new data were created for the purposes of this review. A protocol for this systematic review has been created and registered into OSF (pre-registration #463608).

## References

[1] Jones B., O’Neill B.C., McDaniel L., McGinnis S., Mearns L.O., Tebaldi C. (2015). Future population exposure to US heat extremes. Nat. Clim. Chang..

[2] NOAA National Centers for Enviornmental Information (2023). Monthly Global Climate Report for Annual 2022. https://www.ncei.noaa.gov/access/monitoring/monthly-report/global/202213.

[3] IPCC Intergovernmental Panel on Climate Change (2021). Climate Change 2021: The Physical Science Basis.

[4] Im E.S., Pal J.S., Eltahir E.A.B. (2017). Deadly heat waves projected in the densely populated agricultural regions of South Asia. Sci. Adv..

[5] Pal J.S., Eltahir E.A.B. (2016). Future temperature in southwest Asia projected to exceed a threshold for human adaptability. Nat. Clim. Chang..

[6] Raymond C., Matthews T., Horton R.M. (2020). The emergence of heat and humidity too severe for human tolerance. Sci. Adv..

[7] Russo S., Sillmann J., Sterl A. (2017). Humid heat waves at different warming levels. Sci. Rep..

[8] Levy B.S., Patz J.A. (2015). Climate Change, Human Rights, and Social Justice. Clim. Chang. Glob. Health Hum. Rights.

[9] Hondula D.M., Davis R.E., Saha M.V., Wegner C.R., Veazey L.M. (2015). Geographic dimensions of heat-related mortality in seven U.S. cities. Environ. Res..

[10] Ebi K.L., Capon A., Berry P., Ebi K.L., Capon A., Berry P., Broderick C., de Dear R., Havenith G., Honda Y. (2021). Hot weather and heat extremes: Health risks. Lancet Lond Engl..

[11] Thompson R., Hornigold R., Page L., Waite T. (2018). Associations between high ambient temperatures and heat waves with mental health outcomes: A systematic review. Public Health.

[12] Kuehn L., McCormick S. (2017). Heat Exposure and Maternal Health in the Face of Climate Change. Int. J. Environ. Res. Public Health.

[13] Samuels L., Nakstad B., Roos N., Bonell A., Chersich M., Havenith G., Luchters S., Day L.T., Hirst J.E., Singh T. (2022). Physiological mechanisms of the impact of heat during pregnancy and the clinical implications: Review of the evidence from an expert group meeting. Int. J. Biometeorol..

[14] Syed S., O’Sullivan T.L., Phillips K.P. (2022). Extreme Heat and Pregnancy Outcomes: A Scoping Review of the Epidemiological Evidence. Int. J. Environ. Res. Public Health.

[15] Wells J.C.K. (2002). Thermal Environment and Human Birth Weight. J. Theor. Biol..

[16] Sun S., Weinberger K.R., Spangler K.R., Eliot M.N., Braun J.M., Wellenius G.A. (2019). Ambient temperature and preterm birth: A retrospective study of 32 million US singleton births. Environ. Int..

[17] Chersich M.F., Pham M.D., Areal A., Haghighi M.M., Manyuchi A., Swift C.P., Wernecke B., Robinson M., Hetem R., Boeckmann M. (2020). Associations between high temperatures in pregnancy and risk of preterm birth, low birth weight, and stillbirths: Systematic review and meta-analysis. BMJ.

[18] Rampersad R., Cervar-Zivkovic M., Nelson D.M. (2011). Immunologic Aspects of Pregnancy. The Placenta: From Development to Disease.

[19] Huppertz B. (2011). Vascular Development in the Placenta. The Placenta: From Development to Disease.

[20] Thureen P.J., Trembler K.A., Meschia G., Makowski E.L., Wilkening R.B. (1992). Placental glucose transport in heat-induced fetal growth retardation. Am. J. Physiol-Regul. Integr. Comp. Physiol..

[21] Regnault T.R., de Vrijer B., Galan H.L., Wilkening R.B., Battaglia F.C., Meschia G. (2007). Development and Mechanisms of Fetal Hypoxia in Severe Fetal Growth Restriction. Placenta.

[22] Bonell A., Sonko B., Badjie J., Samateh T., Saidy T., Sosseh F., Sallah Y., Bajo K., Murray K.A., Hirst J. (2022). Environmental heat stress on maternal physiology and fetal blood flow in pregnant subsistence farmers in The Gambia, west Africa: An observational cohort study. Lancet Planet Health.

[23] Krishna U., Bhalerao S. (2011). Placental insufficiency and fetal growth restriction. J. Obstet. Gynaecol. India.

[24] Cowell W., Ard N., Herrera T., Medley E.A., Trasande L. (2023). Ambient temperature, heat stress and fetal growth: A review of placenta-mediated mechanisms. Mol. Cell Endocrinol..

[25] Beltran A.J., Wu J., Laurent O. (2013). Associations of meteorology with adverse pregnancy outcomes: A systematic review of preeclampsia, preterm birth and birth weight. Int. J. Environ. Res. Public Health.

[26] Bekkar B., Pacheco S., Basu R., DeNicola N. (2020). Association of Air Pollution and Heat Exposure With Preterm Birth, Low Birth Weight, and Stillbirth in the US: A Systematic Review. JAMA Netw. Open.

[27] Strand L.B., Barnett A.G., Tong S. (2011). The influence of season and ambient temperature on birth outcomes: A review of the epidemiological literature. Environ. Res..

[28] Dilworth M.R., Sibley C.P. (2013). Review: Transport across the placenta of mice and women. Placenta.

[29] Buse E., Markert U.R. (2019). The immunology of the macaque placenta: A detailed analysis and critical comparison with the human placenta. Crit. Rev. Clin. Lab. Sci..

[30] Ma Y., Hu Y., Ma J. (2023). Animal models of the placenta accreta spectrum: Current status and further perspectives. Front. Endocrinol..

[31] Tricco A.C., Lillie E., Zarin W., O‘brien K.K., Colquhoun H., Levac D., Moher D., Peters M.D., Horsley T., Weeks L. (2018). PRISMA Extension for Scoping Reviews (PRISMA-ScR): Checklist and Explanation. Ann. Intern. Med..

[32] Covidence Systematic Review Software, Veritas Health Innovation, Melbourne, Australia. www.covidence.org.

[33] Andrianakis P., Walker D. (1994). Effect of hyperthermia on uterine and umbilical blood flows in pregnant sheep. Exp. Physiol..

[34] Nakamura H., Nagase H., Ogino K., Hatta K., Matsuzaki I. (2000). Heat Produces Uteroplacental Circulatory Disturbance in Pregnant Rats through Action of Corticotropin Releasing Hormone (CRH). Placenta.

[35] Vähä-Eskeli K., Erkkola R., Irjala K., Uotila P., Poranen A.K., Säteri U. (1992). Responses of placental steroids, prostacyclin and thromboxane A2 to thermal stress during pregnancy. Eur. J. Obstet. Gynecol. Reprod. Biol..

[36] Guo H., Yang Y., Qiao Y., He J., Yao W., Zheng W. (2021). Heat stress affects fetal brain and intestinal function associated with the alterations of placental barrier in late pregnant mouse. Ecotoxicol. Environ. Saf..

[37] Guo H., Liu R., He J., Yao W., Zheng W. (2022). Heat Stress Modulates a Placental Immune Response Associated with Alterations in the Development of the Fetal Intestine and Its Innate Immune System in Late Pregnant Mouse. Front. Physiol..

[38] Dreiling C.E., Carman F.S., Brown D.E. (1991). Maternal Endocrine and Fetal Metabolic Responses to Heat Stress. J. Dairy Sci..

[39] Arora K.L., Cohen B.J., Beaudoin A.R. (1979). Fetal and placental responses to artificially induced hyperthermia in rats. Teratology.

[40] Bell A.W., Wilkening R.B., Meschia G. (1987). Some aspects of placental function in chronically heat-stressed ewes. J. Dev. Physiol..

[41] Dada J.M., Santos M.L., Dani A.P., Dammann C.P., Pinto L., Vieira F.M., Barros F.R. (2023). Placental Development and Physiological Changes in Pregnant Ewes in Silvopastoral and Open Pasture Systems during the Summer. Animals.

[42] Early R.J., McBride B.W., Vatnick I., Bell A.W. (1991). Chronic heat stress and prenatal development in sheep: II. Placental cellularity and metabolism. J. Anim. Sci..

[43] El-Sherbiny H.R., Samir H., El-Shalofy A.S., Abdelnaby E.A. (2022). Exogenous L-arginine administration improves uterine vascular perfusion, uteroplacental thickness, steroid concentrations and nitric oxide levels in pregnant buffaloes under subtropical conditions. Reprod. Domest. Anim..

[44] Galan H.L., Hussey M.J., Barbera A., Ferrazzi E., Chung M., Hobbins J.C., Battaglia F.C. (1999). Relationship of fetal growth to duration of heat stress in an ovine model of placental insufficiency. Am. J. Obstet. Gynecol..

[45] Galan H.L., Hussey M.J., Chung M., Chyu J.K., Hobbins J.C., Battaglia F.C. (1998). Doppler velocimetry of growth-restricted fetuses in an ovine model of placental insufficiency. Am. J. Obstet. Gynecol..

[46] Hensleigh P.A., Johnson D.C. (1971). Heat Stress Effects During Pregnancy. I. Retardation of Fetal Rat Growth*. Fertil. Steril..

[47] Nanas I., Barbagianni M., Dadouli K., Dovolou E., Amiridis G.S. (2021). Ultrasonographic findings of the corpus luteum and the gravid uterus during heat stress in dairy cattle. Reprod. Domest. Anim..

[48] Olivier K., Reinders L.A., Clarke M.W., Crew R.C., Pereira G., Maloney S.K., Wyrwoll C.S. (2021). Maternal, Placental, and Fetal Responses to Intermittent Heat Exposure During Late Gestation in Mice. Reprod Sci..

[49] Padmanabhan R., Al-Menhali N.M., Ahmed I., Kataya H.H., Ayoub M.A. (2005). Histological, histochemical and electron microscopic changes of the placenta induced by maternal exposure to hyperthermia in the rat. Int. J. Hyperth..

[50] Regnault T.R., Orbus R.J., Battaglia F.C., Wilkening R.B., Anthony R.V. (1999). Altered arterial concentrations of placental hormones during maximal placental growth in a model of placental insufficiency. J. Endocrinol..

[51] Silva P.D., Hooper H.B., Manica E., Merighe G.K., Oliveira S.A., Traldi A.D., Negrão J.A. (2021). Heat stress affects the expression of key genes in the placenta, placental characteristics, and efficiency of Saanen goats and the survival and growth of their kids. J. Dairy Sci..

[52] Vähä-Eskeli K.K., Erkkola R.U., Seppänen A., Poranen A.K., Säteri U. (1991). Haemodynamic response to moderate thermal stress in pregnancy. Ann. Med..

[53] Vatnick I., Ignotz G., McBride B.W., Bell A.W. (1991). Effect of heat stress on ovine placental growth in early pregnancy. J. Dev. Physiol..

[54] Walker D.W., Hale J.R., Fawcett A.A., Pratt N.M. (1995). Cardiovascular responses to heat stress in late gestation fetal sheep. Exp. Physiol..

[55] Zhao W., Artaiz O., Iqbal Y., Le H.H., DiGiacomo K., Leury B.J., Fothergill L.J., Furness J.B., Liu F., Green M.P. (2022). Heat stress of gilts around farrowing causes oxygen insufficiency in the umbilical cord and reduces piglet survival. Animal.

[56] Zhao W., Liu F., Bell A.W., Le H.H., Cottrell J.J., Leury B.J., Green M.P., Dunshea F.R. (2020). Controlled elevated temperatures during early-mid gestation cause placental insufficiency and implications for fetal growth in pregnant pigs. Sci. Rep..

[57] Zhao W., Liu F., Marth C.D., Green M.P., Le H.H., Leury B.J., Bell A.W., Dunshea F.R., Cottrell J.J. (2021). Maternal Heat Stress Alters Expression of Genes Associated with Nutrient Transport Activity and Metabolism in Female Placentae from Mid-Gestating Pigs. Int. J. Mol. Sci..

